# Efficient Internet of Things Communication System Based on Near-Field Communication and Long Range Radio

**DOI:** 10.3390/s25082509

**Published:** 2025-04-16

**Authors:** Ting Chai, Dongryool Kim, Seungsoo Shin

**Affiliations:** 1 Department of Computer and Media Engineering, Tongmyong University, Busan 485201, Republic of Korea; me13215992762@gmail.com; 2 College of General Education, Tongmyong University, Busan 485201, Republic of Korea; drkim@tu.ac.kr; 3 Department of Information Security, Tongmyong University, Busan 485201, Republic of Korea

**Keywords:** internet of things, near-field communication, LoRa, Raspberry Pi, communication algorithm optimization, power-efficient

## Abstract

Efficient communication in the Internet of Things (IoT) is essential for enabling smart applications. While NFC excels in near-field device interaction, its limited communication range hinders LoRa’s long-range communication due to its low data throughput. Together with NFC and LoRa technologies, Raspberry Pi is used as a microcontroller (MCU) in this paper to look into how to make near-field and long-distance communication work better together and fix the issue of an imbalance between communication range and energy consumption in the IoT system. By optimizing the communication algorithm and parameter tuning, the power consumption of the system is significantly reduced, and the communication range and data throughput are improved. Our research gives you the technical information you need to make an IoT communication system that works well, uses little power, and has a wide coverage area. This kind of system is good for situations where you need to collect data from a close distance and keep an eye on things from afar. This makes the system more power-efficient and better at communicating, which also makes it easier for users to manage data. It is suitable for a wide range of application scenarios, such as warehousing, healthcare, agriculture, and smart cities.

## 1. Introduction

The rapid development of the Internet of Things (IoT) has brought great potential for innovation and application in various fields, including smart homes, industrial automation, and smart agriculture. In these application scenarios, efficient communication between IoT devices is crucial [1]. Through efficient communication technologies, IoT devices can achieve real-time data transmission, remote monitoring, and automated operations. However, IoT devices typically operate in resource-constrained environments and face challenges such as power consumption, communication distance, and data throughput.

Near-field communication (NFC), as a short-range wireless communication technology, is widely used in applications such as device authentication, data reading, and writing by virtue of its low power consumption, high security, and convenient interaction [2]. However, the communication range of NFC is limited to a few centimeters to a few tens of centimeters, which greatly restricts its application in IoT wide-range environments [3]. On the other hand, LoRa (low-power wide area network technology) is suitable for wide-coverage IoT communication due to its long range and low power consumption, but its lower data transmission rate and latency issues limit applications with high real-time requirements [4]. Despite the advantages of both NFC and LoRa technologies, there are few efficient communication solutions for combining these two technologies in IoT systems. Existing schemes tend to focus on a single communication method that cannot provide long-distance data transmission while satisfying the need for proximity interaction. Also, the balance between NFC and LoRa technologies in terms of data throughput, power consumption, and communication range has not been fully addressed yet, and it is still hard to make switching and working together between the two technologies seamless, especially in IoT devices that do not have a lot of resources [5].

The aim of this paper is to design and optimize an IoT communication system based on a combination of NFC and LoRa, using a Raspberry Pi as a microcontroller (MCU) to coordinate the two communication technologies. The research methodology includes optimizing the communication algorithms for NFC and LoRa, reducing power consumption, increasing the communication range, and improving data throughput through parameter tuning. Experimental results indicate that the system exhibits significant power savings and communication performance enhancement in multiple application scenarios, validating the efficiency and practicality of the combined NFC and LoRa scheme in IoT. This research is unique because it is one of the few systematic studies that programmatically combines NFC with LoRa for IoT communication architecture. Its findings open up new possibilities for smart cities, industrial IoT, and smart agriculture [6].

Currently, the communication technologies of Internet of Things (IoT) systems are mostly based on a single mode, commonly including near-field communication technology (NFC) and long-distance communication technology (LoRa). NFC has the advantages of low power consumption, rapid reading, and high security, which make it suitable for realizing fast and secure data exchange within a short distance, but its communication range is limited and is usually only suitable for close-range applications. In contrast, LoRa technology is widely used in remote monitoring scenarios due to its long-distance communication, low power consumption, and high penetrability, but it lacks flexibility, security, and convenience in close-range data interaction. The limitations of this single communication mode make IoT systems have obvious shortcomings in realizing comprehensive and efficient applications.

To address this issue, this study proposes an IoT architecture that combines both NFC and LoRa technologies to achieve an optimal balance of communication range and energy efficiency. For example, in smart agriculture application scenarios, the use of NFC technology can quickly and conveniently read the detailed information of agricultural equipment or sensor nodes in close proximity, while LoRa is suitable for monitoring and transmitting crop environment information over long distances and with low power consumption. This combination can fully utilize the advantages of both technologies and overcome the challenge of the difficult balance between communication range and energy efficiency of a single technology.

The main contributions of this paper include proposing an IoT framework combining NFC and LoRa; designing adaptive data acquisition frequency control, data differential compression, and data reliable transmission algorithms; constructing hardware prototypes based on Raspberry Pi and ESP32; validating multi-scenario experiments in real-world scenarios; and showing significant performance improvements in energy consumption and communication. By comparing the performance with the existing scheme, it demonstrates the obvious improvement in energy consumption, communication reliability, and system flexibility. This multi-technology fusion solution not only improves the overall performance of the IoT system but also provides a clear, practical reference and theoretical support for similar technology fusion and innovative applications.

## 2. Related Research

In the development of efficient IoT communication systems, near-field communication (NFC) and long-range (LoRa) technologies have gained significant attention due to their complementary characteristics. NFC provides short-range, contact-based communication ideal for close-proximity authentication and data exchange, while LoRa offers low-power, long-range communication suited for IoT applications requiring wide-area connectivity. This section categorizes related research in NFC and LoRa integration into key areas, including energy efficiency, network performance optimization, agricultural applications, authentication mechanisms, and security.

### 2.1. NFC and LoRa in Energy-Efficient Communication

Energy efficiency is a critical consideration in IoT networks, particularly for large-scale deployments involving numerous sensor nodes. Recent studies have leveraged AI and machine learning techniques to optimize energy efficiency and improve resource allocation for LoRaWAN-based IoT networks. For instance, Alkhayyal, M. et al. [7] conducted a systematic review focusing on AI and ML solutions that optimize LoRaWAN’s resource allocation and energy management, while Rosa, R.L. et al. [8] proposed energy-autonomous LoRaWAN sensor nodes using environmental energy harvesting, enabling prolonged operation without external power sources. In addition, energy-efficient designs have been explored for specific applications, such as agricultural irrigation, where different wireless communication technologies, including LoRa, NB-IoT, and ZigBee, have been evaluated for energy consumption and sustainability [9]. These research efforts underscore the importance of optimizing energy use in IoT deployments that utilize both NFC and LoRa for various applications [10,11,12]. Finally, Hanumanthaiah, A. et al. [13] discussed the use of data compression techniques in IoT systems to optimize energy usage by reducing the transmitted data size through compression algorithms, especially for low-cost, low-power (LCLP) communication systems. Al-Kadhim, H.M. et al. [14] proposed adaptive data compression schemes (S-LZW and S-LEC) that help to reduce the power consumption of cloud-based IoT systems.

### 2.2. Network Performance and Data Rate Optimization

LoRa’s low-power wide-area network (LPWAN) characteristics make it suitable for IoT applications; however, optimizing its network performance remains a challenge. A considerable body of research has been dedicated to improving data rate and throughput and reducing latency through adaptive data rate (ADR) optimization. More specifically, Abid Ali Khan, M. et al. [15] talked about a K-means clustering-based spread factor allocation method that works with a backoff algorithm to make electronic shelf label systems faster and less likely to crash. Similarly, Kufakunesu, R. et al. [16] conducted a comprehensive survey on adaptive data rate optimization techniques, discussing their application scenarios and potential improvements to LoRaWAN networks. Multivariate data compression methods using PCA and compressed sampling (CS) in smart metering IoT aim to optimize the bandwidth and increase the data transfer rate [17]. Moreover, we reviewed the application of LoRaWAN across multiple layers, providing insights into improving coverage, handling interference, and optimizing power consumption [18]. These advancements serve as a basis for enhancing communication systems that combine the strengths of NFC for proximity-based operations and LoRa for wide-area communication [19,20,21].

### 2.3. Agricultural Applications of NFC and LoRa Technologies

Agricultural applications represent a significant use case for IoT technologies, where NFC and LoRa have been employed to enhance farm management. NFC is primarily utilized for local authentication and device management, while LoRa supports long-range data transmission, enabling large-scale environmental monitoring. For example, Wan, X. et al. [22] developed an NFC-based agricultural management service system for family farms, allowing efficient, low-cost management of distributed devices and agricultural data tracking. Similarly, Codeluppi, G. et al. [23] proposed a modular LoRaWAN-based smart farming architecture (LoRaFarM), validated through environmental data collection on farms in Italy. Has, M. et al. [24] evaluated data compression in agricultural IoT systems. Huffman coding was used to reduce data traffic and resource usage to optimize the performance of IoT systems in agricultural environments.

Other studies have focused on integrating LoRa with other communication technologies for agricultural IoT networks. For instance, Effah, E. et al. [25] developed and evaluated a multi-hop cluster-based agricultural IoT network using both Bluetooth Low-Energy (BLE) and LoRa, which demonstrated reliability and applicability in rural environments, such as in northern Ghana. This focus on hybrid technologies highlights the growing interest in combining different communication protocols to improve the robustness and scalability of smart farming solutions [11,26,27].

### 2.4. Security and Authentication Mechanisms in IoT

Security is a crucial aspect of IoT communication systems, particularly with the increasing deployment of remote sensors and devices. NFC and LoRa can be effectively combined to address security challenges in IoT deployments. Several studies have explored methods to enhance security using deep learning and blockchain technologies. For instance, Ahmed, A. et al. [28] surveyed the use of deep learning for LoRa radio frequency fingerprinting identification (RFFI) to improve device-level security, thereby ensuring reliable device identification and mitigating impersonation attacks. Additionally, Sidorov, M. et al. [29] proposed a lightweight authentication scheme that represents LoRaWAN nodes as on-chain non-fungible tokens (NFTs), combining blockchain technology with LoRa for secure IoT data management. In terms of mobile payment security, NFC-based protocols have also been improved for IoT environments. Thammarat, C. et al. [30] presented an efficient and secure NFC authentication protocol that ensures a fair exchange during mobile transactions, utilizing lightweight encryption and offline session key generation techniques. These advancements demonstrate the feasibility of integrating NFC and LoRa to create secure IoT systems, addressing both short-range and long-range communication needs effectively [31,32].

### 2.5. Specialized Applications of NFC and LoRa Integration

The combination of NFC and LoRa has also been applied in specialized domains such as underground sensor networks and food safety. In Xue-fen, W. et al. [33], a smartphone-based LoRa in-soil propagation measurement system called “LoRa Wizard” was developed to monitor underground environments. The system demonstrated low-cost, high-flexibility potential for agricultural and accident prevention applications. Similarly, Crul, S. et al. [34] presented an IoT solution combining NFC for near-field identification with LoRa for long-range communication to improve safety and traceability in food and feed delivery. This combination highlights the versatility of NFC and LoRa in addressing diverse IoT application needs [12,35,36].

### 2.6. Surveys and Reviews on LoRa and NFC in IoT

A substantial portion of the research in this area has been dedicated to providing comprehensive reviews of existing technologies and their applications. Shanmuga Sundaram, J.P. et al. [35] presented a survey focusing on the technological challenges, solutions, and open issues in LoRa networking, addressing both physical and MAC layer challenges. Similarly, Bonilla, V. et al. [36] conducted a systematic literature review summarizing LoRaWAN applications in agriculture, healthcare, and environmental monitoring, providing insights into the features and technical requirements of LoRa devices. Guberovic, E. et al. [37] studied various compression algorithms for IoT sensor nodes, focusing on efficient communication methods with low power consumption and low complexity, and explored various techniques related to LoRa and NFC integration in-depth. These reviews not only outline the technological progress but also point out gaps and future research opportunities for integrating NFC and LoRa, particularly in areas like adaptive optimization, energy-efficient communication, and secure authentication. This integrated understanding lays the foundation for the development of efficient IoT communication systems that harness the strengths of both NFC and LoRa technologies [10,19,20,32].

### 2.7. Comparison with Existing NFC–LoRa Integration Studies

Griese, M.G. et al. [38] proposed a vehicle identification system based on RFID and LoRa, which utilizes LoRa to achieve the long-distance transmission of vehicle information to meet the needs of Intelligent Transportation Systems (ITSs), but their research focuses on the evaluation of communication performance in urban scenarios, and does not explore power consumption management and algorithm optimization in-depth. Deng, F. et al. [39] proposed a soil environment monitoring system that buries RFID sensors underground and collects data over long distances through LoRa modules on inspection vehicles. RFID sensors are buried in the ground and collect data over long distances through LoRa modules on inspection vehicles, focusing on solving long-distance transmission and energy harvesting in large-area agricultural environments, but it only did basic experiments on the sensor hardware design and the communication success rate, and did not deal with system-level data aggregation or algorithmic optimization. Sendra, S. et al. [40] developed a LoRa- and NFC-based smart baby care box system, which achieves the long-distance uploading of medical data and near-field doctor identification. Although it also takes into account near-field data interaction and long-distance transmission, it does not involve differential data aggregation, power consumption optimization, or transmission reliability enhancement algorithms, and mainly focuses on basic performance evaluation in a laboratory environment.

## 3. Efficient IoT Communication Model Design

### 3.1. Overview

**Figure 1:**
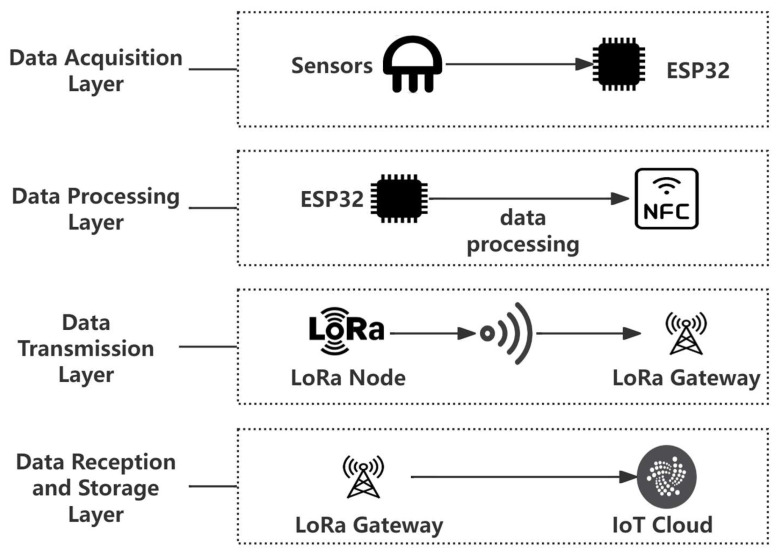
Communication system structure diagram.

The proposed communication model aims to enhance the efficiency and reliability of data transmission in IoT devices by leveraging near-field communication (NFC) technology. The system integrates NFC with LoRa communication to achieve both short-range and long-range data transfer while optimizing power consumption. Concerning the Internet of Things Device Node, the system is equipped with sensors, an ESP32 microcontroller that serves as the primary controller, an NFC module, and a LoRa module. The tasks assigned to this node include data collection, processing, temporary storage, and transmission. The NFC tag serves as a data storage medium for short-range communication, enabling rapid data writing and reading between the ESP32 and the LoRa module. The LoRa gateway receives data supplied through the LoRa module and relays it to the IoT cloud platform. The IoT cloud platform offers storage, processing, and analysis of the acquired data. The communication system is structured to facilitate seamless data flow from the sensor nodes to the cloud platform. The architecture depicted in Figure 1 comprises four components.

First is the Data Acquisition Layer, where sensors connected to the ESP32 microcontroller collect environmental data such as temperature, humidity, and pressure. Second is the Data Processing Layer, in which the ESP32 processes the raw sensor data, applying algorithms for frequency adjustment, data compression, and error detection, before writing the processed data to the NFC tag. Third is the Data Transmission Layer, where the LoRa module reads data from the NFC tag and transmits it to the LoRa gateway over long distances. Finally, the Data Reception and Storage Layer involves the LoRa gateway receiving the data and forwarding it to the IoT cloud platform, where the data are stored and made available for further analysis.

In order to clarify the reasons for choosing Raspberry Pi as the edge computing and LoRa gateway platform for this system and to systematically compare it with other common microcontrollers and embedded platforms, we have considered the following dimensions:

First, in terms of the operating system and software support, Raspberry Pi is equipped with the Debian-based Raspbian operating system, which supports the full Linux user space and system-level functionality and facilitates the integration of network stacks, databases, graphical interfaces, and high-level programming languages, which is especially beneficial for scenarios where you need to locally execute data-processing algorithms, visualize renderings, or run LoRa network stacks. In contrast, microcontroller platforms such as Arduino and STM32 typically run bare-metal programs or lightweight RTOSes that lack support for file systems, multitasking, and complex network services, limiting functionality and software maintenance.

Second, from the perspective of hardware performance, Raspberry Pi is equipped with a high-frequency multi-core processor and large-capacity memory, which provides sufficient computing power for the local operation of complex data processing algorithms, database management, data compression, and graphic display, which is more difficult to be realized on an MCU (such as STM32) with limited computing resources.

Third, the richness of interface resources is a major advantage of Raspberry Pi. Raspberry Pi integrates multiple interfaces such as USB, HDMI, GPIO, SPI, I^2^C, UART, etc., and supports the connection of cameras, monitors, sensor modules, NFC readers, LoRa modules, etc., which is highly scalable and compatible. In contrast, platforms such as Arduino and STM32 are limited in the number of peripherals and bandwidth and often require additional interface expansion chips or customized designs, increasing the complexity of hardware development and debugging.

However, it should be noted that the core research and innovation of this system does not depend on the hardware characteristics of a specific platform but lies in the design of the cooperative communication mechanism between NFC and LoRa and the optimization of its related algorithms, including the adaptive control of the data acquisition frequency, differential compression and aggregation, the packet loss detection and retransmission mechanism, etc. Therefore, this system can be deployed on STM32, ESP32, or other microcontroller platforms with certain processing capability and communication interface, with good cross-platform adaptability and portability. Mainly for the convenience of rapid prototyping, algorithm verification, system demonstration, and multi-functional integration, it provides a good foundation for subsequent practical deployment and platform migration while taking into account the development efficiency and system functions.

### 3.2. Model Workflow

#### 3.2.1. Data Flow Process

The process begins with sensor data collection, where sensors are connected to the ESP32 collect data ($S_{t}$) at time t. The collected data include various environmental parameters necessary for the application. Second, in the data processing and storage phase, the ESP32 computes the rate of change ($∆S_{t}$) using Algorithm A, which is an adaptive data acquisition and transmission frequency control algorithm. Based on $∆S_{t}$, ESP32 adjusts the data acquisition frequency $(f_{t}$). The processed data are then written to the NFC tag for temporary storage. The rate of change ($∆S_{t}$) is defined as the absolute difference between the current data and the previous data point.

**Figure 2:**
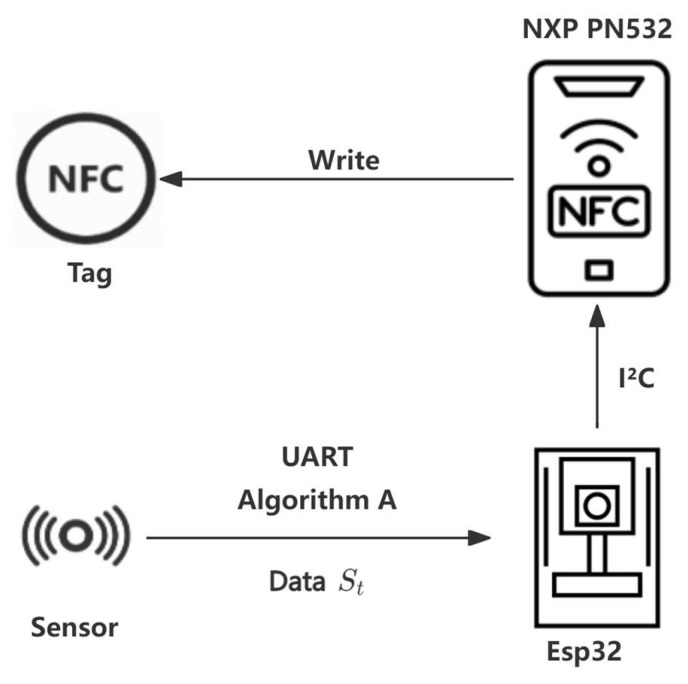
NFC tag writing diagram.

Third, during the NFC tag writing phase, ESP32 communicates with the NFC module over SPI or I^2^C protocols. The current data ($S_{t}$) is written to the NFC tag using standard NFC protocols, forming a data queue by storing multiple data points. Figure 2 illustrates the NFC tag writing process. Fourth, in the data reading and compression phase, the LoRa module, equipped with an NFC reader, reads the data from the NFC tag. Algorithm B, known as the Compression and Aggregation Transmission Algorithm, is used to compress and aggregate the data, effectively reducing the data size for efficient transmission.

Fifth, in the data packet construction phase, a data packet is constructed that includes sequence numbers and checksum values, as specified by Algorithm C (Data Packet Loss Detection and Retransmission Algorithm). This packet structure helps ensure data integrity and facilitates error detection. Sixth, during LoRa data transmission, the compressed data packet is transmitted over the LoRa network to the LoRa gateway. LoRa’s long-range communication capabilities enable transmission over several kilometers. Seventh, during data reception and validation, the LoRa gateway receives the data and forwards it to the IoT cloud platform, where the data are validated. This includes checking the sequence continuity and verifying checksum correctness. Eighth, in the error handling and retransmission phase, if any data loss or corruption is detected, the cloud platform sends a retransmission request back to the IoT device via the LoRa network. The device then retransmits the required data packets, ensuring data completeness. Finally, in the data decompression and storage phase, upon successful reception, the cloud platform decompresses the data using the initial value and the differential sequence to restore the original sensor data ($S_{t}$). The restored data are then stored in the database for further analysis.

#### 3.2.2. Workflow Diagram Description

**Figure 3:**
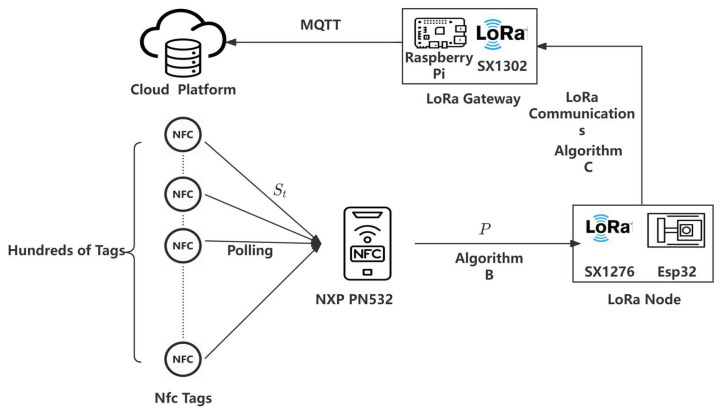
LORA remote communication flowchart.

Figure 3 illustrates the LoRa distant communication method, and the comprehensive workflow depicted in Figure 2 and Figure 3 may be encapsulated as follows: First, in the near-field communication (NFC) phase, ESP32 collects sensor data, processes it, applies frequency adjustment using Algorithm A, and writes the processed data to the NFC tag. In the second phase, LoRa remote communication, the LoRa module reads data from the NFC tag, compresses and aggregates it using Algorithm B, constructs a data packet with error detection utilizing Algorithm C, and transmits the packet via LoRa. Upon reception, the data undergoe validation, with possible retransmission if errors are detected, followed by decompression and storage on the cloud platform.

### 3.3. Algorithm Design

#### 3.3.1. Adaptive Data Acquisition and Transmission Frequency Control (Algorithm A)

This algorithm dynamically adjusts the data acquisition and transmission frequency based on the rate of change in sensor data. The goal is to increase the frequency during significant data changes to ensure real-time monitoring and decrease it during stable periods to conserve energy. The data rate of change is determined as specified in Equation (1).

(1) $$∆S_{t}=|S_{t}-S_{t-1}|$$

where *t* is used to track the time series of data collection, ensuring that each collection has a corresponding time stamp. Where $S_{t}$ and $S_{t-1}$ are used to calculate the degree of variation in the data to help the algorithm determine if the collection frequency needs to be adjusted. Where $∆S_{t}$ denotes the rate of change in the data at time point *t*, i.e., the absolute difference between the current data and the previous data.

Frequency adjustment is determined by Equation (2).

(2) $$f_{t}=\left\{\begin{array}{c} f_{max},\;\;\;if\;\;∆S_{t}\geq \;\theta_{∆}\\ f_{min},\;\;\;if\;\;∆S_{t}<{\;\theta }_{∆} \end{array}\right.$$

A predefined threshold $\theta_{∆}$ is set to evaluate significant changes. The implementation steps are divided into initialization and data acquisition loop. During the initialization phase, the maximum frequency ($f_{max}$), minimum frequency ($f_{min}$), and threshold ($\theta_{∆}$) are set, followed by initializing the previous data value ($S_{t-1}$). In the data acquisition loop, the current data ($S_{t}$) are collected, and the rate of change ($∆S_{t}$) is computed. Based on $∆S_{t}$ and the threshold $\theta_{∆}$, the data collection frequency ($f_{t}$) is adjusted accordingly. The previous data ($S_{t-1}$) are then updated with the current data ($S_{t}$), and the system waits for the next acquisition cycle, as determined by $f_{t}$.

#### 3.3.2. Compression and Aggregation Transmission (Algorithm B)

This algorithm reduces the volume of data transmitted by compressing and aggregating multiple data points. It employs differential encoding and data aggregation to create compact data packets.

The differential code is determined by Equation (3).

(3) $${D_{t}=S}_{t}{-S}_{t-1}$$

The data aggregation formula is determined by Equation (4).

(4) $$P=\left\{D_{t-N+1,}D_{t-N+2},\ldots ,D_{t}\right\}$$

For a window size of *N*, the positive integers specify the number of differential data points to be aggregated. The packet *P* contains *N* differential data from the time point *t − N +* 1 to *t*, as well as the initial value $S_{t-N}$, allowing the receiver to restore the complete data sequence. The initial value $S_{t-N}$ is stored to reconstruct the original data at the receiver. The subsequent implementation steps are as follows: First, *N* data points are collected from the NFC tag. Next, compression is performed by computing the differential values ($D_{t}$). Following this, packet formation takes place by combining $S_{t-N}$ and the differential sequence into a packet (*P*). The packet is then transmitted via the LoRa module. Finally, at the receiver, decompression is conducted by using $S_{t-N}$ and $D_{t}$ to reconstruct the original data $S_{t}$.

#### 3.3.3. Data Packet Loss Detection and Retransmission (Algorithm C)

This algorithm enhances data transmission reliability by incorporating sequence numbers and checksums into data packets. It enables the receiver to detect missing or corrupted packets and request retransmission.

The packet structure is determined by Equation (5).

(5) $$Packet=\left\{Seq_{Num},Data,Checksum\right\}$$

where Seq_Num represents the sequence number, an integer value used to uniquely identify each data packet. It helps the receiver detect lost or out-of-order packets. Data represents the data, which is the actual content to be transmitted, such as sensor measurements. Checksum represents the checksum, used to verify the integrity of the data packet and detect errors that may have occurred during transmission.

The checksum calculation is determined by Equation (6).

(6) Checksum=CRC(Seq_Num||Data)

where CRC is the Cyclic Redundancy Check function, a method used to compute a checksum based on the input data, which helps in detecting errors in data transmission. || denotes concatenation, meaning that Seq_Num and Data are concatenated together to form a single input for the CRC function. The subsequent steps for implementation are as follows. At the sender side, a sequence number (Seq_Num) is assigned to each packet, and a checksum is computed using CRC. Then, the packet is constructed and sent. At the receiver side, the sequence number is verified for continuity, and the checksum is computed and compared. If discrepancies are found, the receiver requests retransmission, specifying the missing sequence numbers. During retransmission, the sender resends the requested packets to ensure data integrity.

### 3.4. Detailed System Workflow

#### 3.4.1. Data Acquisition and NFC Tag Writing

Sensor Interface: Sensors collect data $S_{t}$ and send it to ESP32 via the appropriate interfaces (e.g., analog inputs, I^2^C, SPI). Processing with Algorithm A: ESP32 processes $S_{t}$, computes ${∆S}_{t}$, and adjusts $f_{t}$. NFC Tag Writing: ESP32 communicates with the NFC module over SPI or I^2^C. Data $S_{t}$ is written to the NFC tag using standard NFC protocols. Multiple data points can be stored on the NFC tag, forming a data queue.

#### 3.4.2. Data Reading and Transmission via LoRa

NFC Data Reading: The LoRa module, integrated with an NFC reader, reads the stored data from the NFC tag. Reading is performed at intervals optimized for energy efficiency and data timeliness. Data Compression with Algorithm B: The read data are compressed using differential encoding. A data packet P is formed, including the initial value and the differential sequence. Packet Construction with Algorithm C: A sequence number Seq_Num is assigned. The checksum Checksum is calculated. The final packet includes all necessary headers and payloads. LoRa transmission: The packet is transmitted over the LoRa network. LoRa modulation parameters (e.g., spreading factor, bandwidth) are configured for optimal range and reliability.

#### 3.4.3. Data Reception and Processing on the Cloud Platform

Data Reception: The LoRa gateway receives the data packet and forwards it to the cloud platform via the internet. Data Validation: The cloud platform checks the sequence number for continuity and recalculates the checksum to verify data integrity. Error Handling: If errors are detected, the cloud platform sends a retransmission request to the IoT device via the LoRa network. The device retransmits the specified data packets. Data Decompression: Upon successful reception, the cloud platform decompresses the data using the initial value and differential sequence and then reconstructs the original sensor data $S_{t}$.

**Figure 4:**
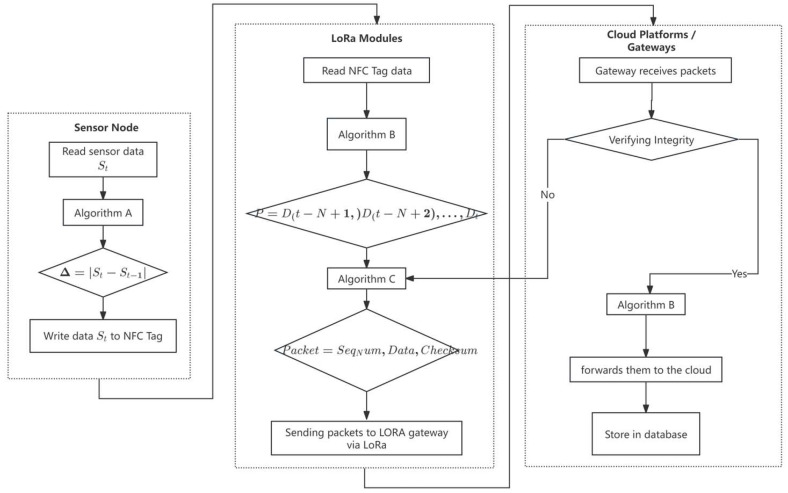
NFC-LoRa integrated flowchart.

In order to more intuitively help readers understand the technical contributions of the IoT communication system proposed in this thesis, we design a comprehensive system flowchart, which demonstrates in detail the key steps in developing the system, including the following: the sensor nodes carry out the read/write exchanges of data through near-field communication (NFC); the LoRa module realizes the long-distance transmission process of the data, which covers the construction of data packets, compression and aggregation strategies, as well as the retransmission mechanism in the event of data loss; and the server side clearly demonstrates the validation, decompression, storage, and interpretation and analysis process of the received data. Through this flowchart, readers can clearly trace the life cycle of data in the whole system and deeply understand the synergistic mechanism and data flow process between various modules of the system. The integrated flowchart is shown in Figure 4.

### 3.5. NFC–LoRa Interference Consideration

In the designed IoT communication system, two primary wireless communication protocols are employed: NFC operating at 13.56 MHz and LoRa typically operating at frequencies such as 433 MHz, 868 MHz, or 915 MHz. Given the significant frequency gap between NFC and LoRa, direct interference at the physical hardware level is inherently limited. Specifically, the NFC antenna and LoRa antenna have different design characteristics, impedance matching considerations, and physical dimensions due to their operational frequency differences, further reducing the likelihood of cross-interference.

Nonetheless, potential interference may exist at the software or protocol layer, particularly when both NFC and LoRa modules share the same microcontroller unit (MCU). To mitigate this, careful task scheduling at the MCU level is essential. This can be managed by avoiding concurrent intensive communication tasks, assigning different interrupt priority levels, or utilizing timer-based staggered scheduling strategies to ensure NFC and LoRa operations occur at distinct time intervals.

Additionally, if deemed necessary by system performance evaluation, a basic communication time-slot management strategy can be implemented at the system level. For instance, the MCU can be programmed to ensure that when the LoRa module is actively transmitting or receiving, the NFC module operates in passive listening mode or temporarily suspends active communication tasks. Such arrangements effectively prevent resource contention on the MCU and minimize unnecessary latency or communication errors.

In summary, by clearly delineating communication frequencies, antenna configurations, and implementing intelligent software-based scheduling, potential NFC–LoRa interference within the proposed IoT system architecture can be effectively managed or completely avoided.

### 3.6. Extension to Uncertain or Dynamic Environments

In practical wireless communication scenarios, there are inherent uncertainties such as channel estimation errors, unpredictable user mobility, and varying environmental conditions. The proposed IoT communication architecture, combining NFC and LoRa technologies, can be extended to address these complex and dynamic scenarios by incorporating adaptive mechanisms to enhance system robustness and reliability.

#### 3.6.1. Adaptive Algorithm Extension

To address uncertainties such as channel fading and estimation errors, the adaptive frequency control (Algorithm A) can be further augmented by incorporating real-time network measurement feedback, including Received Signal Strength Indicator (RSSI) and Signal-to-Noise Ratio (SNR). This enhancement allows the system to dynamically adjust data acquisition and transmission strategies based on instantaneous channel quality, thus optimizing performance and reducing transmission errors under fluctuating conditions. For instance, a lower SNR or a sudden drop in RSSI can trigger an automatic increase in transmission power or a reduction in spreading factor for LoRa communications, ensuring data integrity even when wireless channels degrade.

#### 3.6.2. Reliability Enhancement Strategies

Considering user mobility and unpredictable user locations, the LoRa communication layer can integrate advanced reliability mechanisms, such as multi-hop communication with dynamic hop-count limitations or automatic gain control (AGC). Multi-hop routing strategies can extend effective communication coverage and adapt to changing node distributions. Additionally, AGC mechanisms can dynamically adjust transmission power and receiver sensitivity, ensuring consistent connectivity as users or sensor nodes move unpredictably within the coverage area.

By incorporating these adaptive strategies, the system effectively mitigates the adverse effects of channel uncertainty and user mobility, enhancing overall system robustness and reliability in dynamic and uncertain IoT deployment scenarios.

## 4. Evaluation and Results

To validate the effectiveness and superiority of the proposed efficient IoT communication model based on NFC and LoRa, comprehensive experiments and evaluations were conducted. This section details the experimental setup, evaluation methodology, simulated experimental data analysis, and comparative analysis with the existing literature to quantify the improvements achieved by our model.

### 4.1. Experimental Design and Implementation

#### 4.1.1. Objectives

The main objectives of the experimental evaluation are threefold. The first is to perform feasibility validation to demonstrate the practical feasibility of the proposed communication model and algorithms in a real-world scenario. Secondly, performance evaluation is conducted to assess the performance of the model in terms of data transmission efficiency, energy consumption, and reliability. Finally, the performance of the model is evaluated by comparative analysis; we compare the performance of our model with existing solutions and quantify the improvements.

#### 4.1.2. Experimental Setup

**Figure 5:**
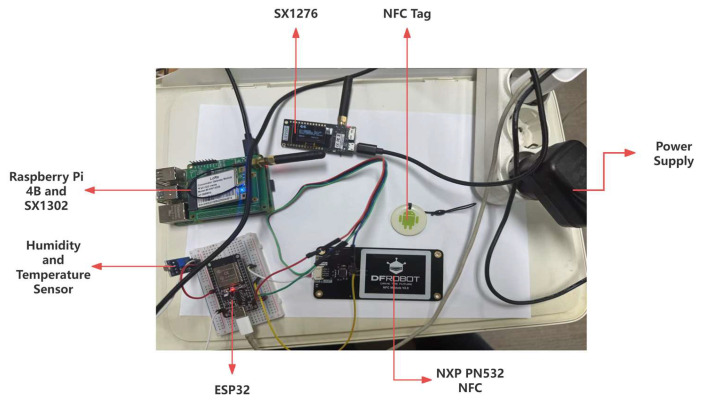
Prototype setup of IoT node and LoRa gateway.

Our system comprises sensor nodes equipped with ESP32 microcontrollers, which serve as the core controllers for managing data collection and communication. These nodes incorporate temperature and humidity sensors like DHT22 for collecting environmental data and NFC modules such as PN532 to facilitate NFC tag writing and reading. For long-range data transmission, they utilize LoRa modules like SX1276. An INA226 power monitoring chip is integrated to monitor power consumption, and NFC readers are incorporated with ESP32 for enhanced data collection. The collected data are transmitted to gateway devices, including a LoRa gateway that receives data from the sensor nodes and forwards it to a Raspberry Pi acting as a server for data processing and storage. Power supply units are employed throughout the system to provide stable power to all devices. As shown in Figure 5, the prototype setup includes the IoT node based on ESP32 with SX1276 and the LoRa gateway using the SX1302 chip.

Our software environment encompasses firmware development for ESP32, implemented in C++ and incorporating algorithms A, B, and C. On the server side, we utilize Raspbian as the operating system on a Raspberry Pi, with Python 3.10.9 employed for data processing. Data storage is managed using databases like MySQL or MongoDB, and data visualization is facilitated through tools such as Matplotlib 3.7.1 and Grafana 9.4.7. For communication, we adhere to standard NFC Forum protocols for NFC interactions, use the LoRaWAN protocol for LoRa communication, and implement network protocols like TCP/IP and MQTT for data transmission. Our experimental environment includes both indoor settings for controlled initial testing and outdoor settings to evaluate long-range communication and assess interference in real-world conditions.

To facilitate comparison with common LoRaWAN unoptimized scenarios, we define a baseline without adaptive frequency control (Algorithm A), without differential compression (Algorithm B), and without packet retransmission detection (Algorithm C). Its main parameter settings refer to the conventional LoRa node configurations in the literature [21], i.e., a fixed sampling frequency (every 5 min), a fixed transmission power (14 dBm), and a minimal CRC checksum enabled only at the hardware level.

The proposed model, on the other hand, introduces Algorithm A/B/C on top of the above baseline to optimize the sampling and compression process under the same hardware platform. This design allows us to directly compare the differences in important metrics under the same hardware conditions “with and without optimization” and also aligns with the fixed-frequency model used in common LoRa studies, such as those in the literature [21], thus ensuring the comparability and representativeness of the baseline.

#### 4.1.3. Experimental Procedures

The system assembly involved integrating the ESP32 microcontroller with temperature and humidity sensors, an NFC module, a LoRa module, and the INA226 power monitoring chip to create the sensor nodes. Simultaneously, the LoRa gateway and the Raspberry Pi server were configured to handle data reception and processing.

For algorithm implementation, Algorithm A (Adaptive Frequency Control) was deployed on ESP32 to adjust the data acquisition frequency based on variations in sensor data. Algorithm B (Data Compression and Aggregation) was implemented to compress and aggregate data before transmission, enhancing efficiency. To ensure reliable data transmission, Algorithm C (Packet Loss Detection and Retransmission) was incorporated to detect lost packets and initiate retransmission when necessary. Parameter configuration was carefully set to optimize performance. The data variation threshold ($\theta_{∆}$) was established at 0.5 °C for temperature changes. The maximum frequency ($f_{max}$) was set to one acquisition per minute, while the minimum frequency ($f_{min}$) was configured for one acquisition every five minutes. The aggregation window size (NN) was set to 10 data points to balance data granularity and transmission efficiency. During data collection and transmission, the sensor nodes initiated data acquisition. ESP32 processed the collected data and wrote it to the NFC tag. An NFC reader then retrieved the processed data from the NFC tag. The compressed and aggregated data packets were transmitted via the LoRa module to the gateway, ensuring efficient long-range communication.

Upon data reception and processing, the LoRa gateway forwarded the received data to the Raspberry Pi server. Server side applications validated and decompressed the data before storing it in the database. Throughout this process, power consumption data were recorded using the INA226 chip, providing insights into the system’s energy efficiency.

Performance metrics—including data transmission times, packet loss rates, energy consumption, and data acquisition frequencies—were meticulously recorded. These metrics were essential for assessing the system’s performance and identifying areas for improvement. The experiments were repeated under varying environmental conditions, such as different distances, obstacles, and interference levels. By adjusting algorithm parameters, we observed changes in performance, which aided in fine-tuning the system for optimal operation in both controlled and real-world scenarios.

#### 4.1.4. Experimental Environment Details

This section provides comprehensive details regarding the experimental setup, including specific hardware models, chip specifications, sensor accuracy, and software configurations used in the experiments.

The experimental system’s main hardware includes an ESP32-WROOM-32 microcontroller unit (MCU) with a dual-core Xtensa LX6 processor running at 240 MHz and built-in Wi-Fi and Bluetooth support; an NXP PN532 NFC module running at 13.56 MHz with SPI and I^2^C interfaces, allowing a read/write range of up to 5 cm; a Semtech SX1276 LoRa communication module that works with frequencies from 137 MHz to 1020 MHz and has a programmable RF transmit power range of −4 dBm to +20 dBm and a receiver sensitivity of down to −148 dBm; and a Semtech SX1302 LoRa gateway that can receive multiple channels and multiple data rates at the same time, improving network capacity and handling packet collisions. A Raspberry Pi 4 Model B with a Broadcom BCM2711 quad-core Cortex-A72 CPU running at 1.5 GHz and 4 GB of RAM was also used as the main computer for processing, storing, and analyzing data. The system uses a Texas Instruments INA226 chip for accurate power tracking. This chip can measure voltage and current with a high level of accuracy (±0.1%) and can handle sampling rates of up to 500 samples per second.

Tests were conducted within a laboratory space (approximately 100 m^2^), characterized by typical office furniture and walls made of standard gypsum board. The LoRa module and gateway were positioned at distances ranging from 5 m to 30 m to evaluate short-range communication efficiency and signal attenuation due to indoor obstacles. Tests were executed in open outdoor areas and semi-obstructed scenarios (including buildings and vegetation) with distances ranging from 100 m up to 1 km to assess the long-range capabilities and reliability of the LoRa communication under realistic environmental conditions.

Key LoRa configuration parameters during the testing process were fixed to ensure consistent performance evaluation. The frequency channel is set to a fixed frequency channel of 868 MHz (Europe). Bandwidth is fixed at 125 kHz to achieve the best trade-off between the range and data rate. The configuration of diffusion coefficients SF7 to SF12 was tested, with SF10 primarily used for balancing range and reliability.

### 4.2. Evaluation Methodology

Our performance metrics focused on data transmission efficiency, energy consumption, reliability, and adaptability. Data transmission efficiency was evaluated through the compression ratio and transmission latency—the average time taken for the data to travel from the sensor node to the server. Energy consumption was assessed by measuring the average power consumption in milliwatts (mW) for each module and calculating energy savings. The reliability metrics included the packet loss rate and the retransmission success rate, which is the percentage of successfully retransmitted packets. Adaptability was measured by the frequency adjustment response time, indicating how quickly the system adjusted the data acquisition frequency after detecting data variation. For data analysis methods, we used statistical analysis involving mean values, standard deviations, and confidence intervals to assess the system’s performance. Comparative analysis was conducted by comparing our results with baseline models and findings from other literature. Visualization tools like graphs and charts were employed to illustrate performance trends effectively.

### 4.3. Simulated Experimental Data Analysis

#### 4.3.1. Data Transmission Efficiency

**Figure 6:**
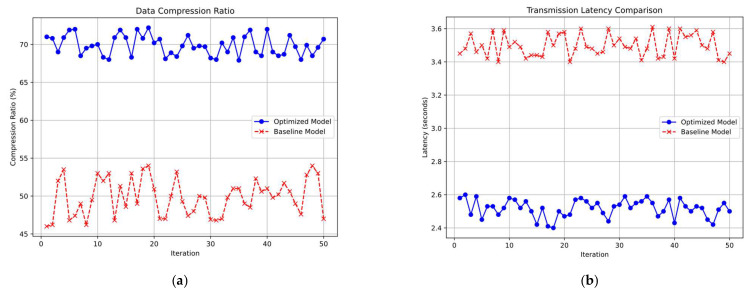
Comparison of data compression and transmission delay performance. (**a**) Data Compression Ratio Comparison: Optimized Model vs. Baseline Model. (**b**) Transmission Latency Comparison: Optimized Model vs. Baseline Model.

We achieved an average compression ratio of 70%, significantly reducing the data size transmitted. This efficiency is attributed to Algorithm B, which effectively compresses and aggregates data before transmission, enhancing overall transmission efficiency. The average transmission latency was reduced to 2.5 s from the 3.5 s observed in baseline models. The reduction in latency is due to the smaller data packets and optimized transmission frequency, which allow for faster data delivery. Algorithm B played a crucial role in enhancing transmission efficiency. By significantly reducing data size, it minimized the bandwidth required for transmission and contributed to lower latency. The combination of transmitting smaller data packets and optimizing transmission frequency led to a noticeable decrease in latency. This improvement enhances the system’s responsiveness and data delivery speed. As shown in Figure 6, Figure 6a illustrates the average compression ratio for both the proposed model and the baseline model, while Figure 6b presents the corresponding average transmission delay for each model.

#### 4.3.2. Energy Consumption

**Figure 7:**
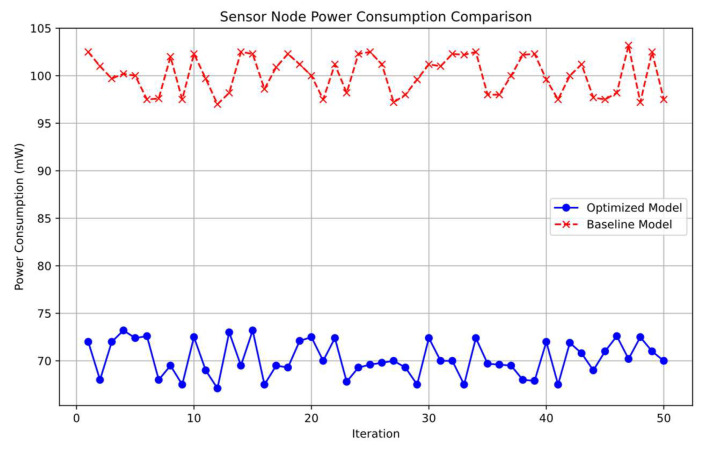
Sensor node power consumption comparison: optimized model vs. baseline model.

In the baseline model, the average power consumption of a sensor node was 100 mW. Our proposed model reduced this to 70 mW, resulting in a 30% energy saving. This significant reduction is primarily due to the implementation of adaptive frequency control (Algorithm A), which minimizes unnecessary data acquisition and transmission. The power consumption of the LoRa module was reduced by 25% because of fewer transmissions. By sending data only when necessary, the system conserves energy without compromising performance. We achieved an overall energy saving of 28% compared to the baseline. The use of the INA226 power monitoring chip allowed for dynamic adjustments to optimize energy usage, further enhancing the system’s energy efficiency. As shown in Figure 7, we compare the sensor node power consumption for both the proposed optimized model and the baseline model across multiple iterations. It can be observed that the optimized model maintains a consistently lower power consumption level.

#### 4.3.3. Reliability

**Figure 8:**
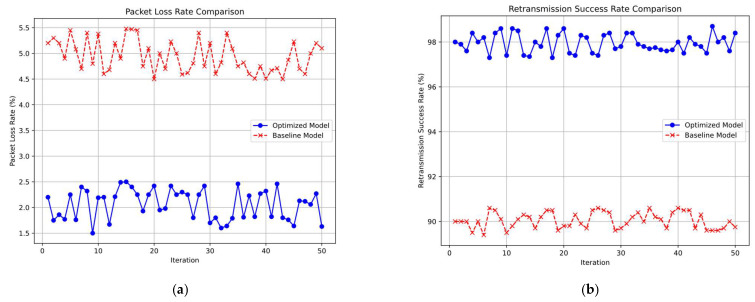
Comparison of packet loss rate and retransmission success rate performance. (**a**) Packet Loss Rate Comparison: Optimized Model vs. Baseline Model. (**b**) Retransmission Success Rate Comparison: Optimized Model vs. Baseline Model.

The packet loss rate was reduced from 5% in the baseline model to 2% in our proposed model. This improvement highlights the system’s enhanced reliability in data transmission. We achieved a 98% success rate in packet retransmissions. This high success rate is due to Algorithm C, which effectively detects and retransmits lost packets, ensuring data integrity. The use of the INA226 chip for power monitoring allowed for dynamic energy optimization. Additionally, Algorithm C improved the system’s robustness by maintaining low packet loss even under adverse conditions, such as interference or obstacles. As shown in Figure 8, Figure 8a presents a comparison of the packet loss rate between the proposed model and the baseline model, while Figure 8b illustrates the corresponding retransmission success rate.

#### 4.3.4. Adaptability

**Figure 9:**
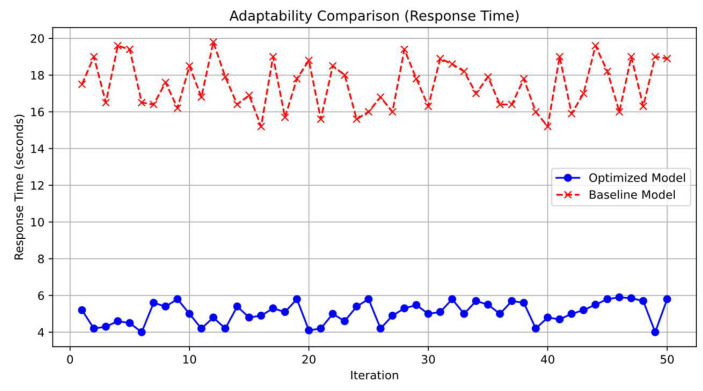
Adaptability comparison (response time): optimized model vs. baseline model.

The system demonstrated an average frequency adjustment response time of 5 s. This quick response ensures that the system adapts promptly to changing environmental conditions. During sudden temperature changes, the system increased the data acquisition frequency from once every 5 min to once per minute within 1 min. This dynamic scaling allows for more timely data collection when significant environmental variations occur. The model quickly adapted to environmental changes, ensuring timely and relevant data collection. This responsiveness demonstrates the system’s enhanced flexibility and its ability to meet varying application demands effectively. As shown in Figure 9, we compare the adaptability of the proposed optimized model against the baseline model by examining their response times across multiple iterations. It can be observed that the optimized model consistently achieves lower response times, indicating superior adaptability.

#### 4.3.5. Comparison with Outdoor Environment

The baseline model is characterized by fixed data acquisition and transmission frequencies, lacking data compression or aggregation, and without any packet loss detection or retransmission mechanisms. In contrast, our proposed model demonstrates significant efficiency gains by outperforming the baseline in all key metrics. The adaptive algorithms contribute to substantial energy savings, enhancing energy efficiency. Additionally, the implementation of packet loss detection and retransmission improves data integrity, resulting in enhanced reliability.

In Table 1, we present a comparative analysis of the unoptimized and optimized approaches across four key metrics (data transmission efficiency, energy consumption, communication reliability, and adaptability) in both outdoor and indoor environments. The results demonstrate clear advantages of the optimized model, including marked increases in data transmission efficiency (up to 59.9% outdoors and 38.9% indoors), significant reductions in energy consumption (up to 22.2% outdoors and 26.4% indoors), notable improvements in communication reliability (up to 10.8% outdoors and 6.2% indoors), and substantial decreases in response times (up to 63.4% outdoors and 72.4% indoors). Overall, these findings highlight the robustness and effectiveness of the optimized approach under various operational conditions.

**Figure 10:**
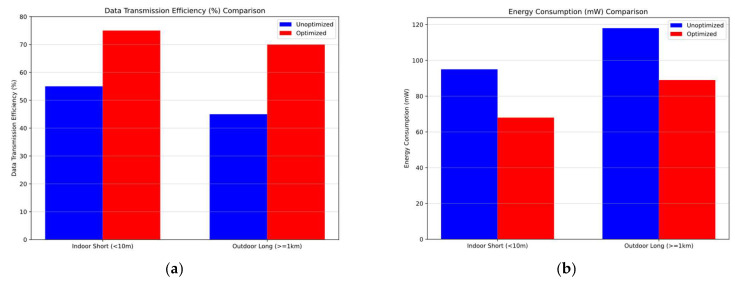

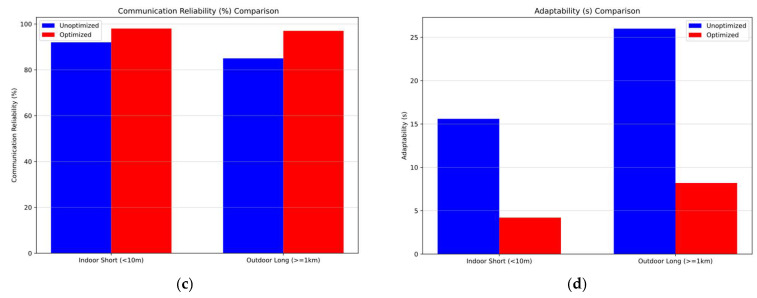
Comparison of data transmission efficiency, energy consumption, communication reliability, and adaptability performance. (**a**) Data Transmission Efficiency (%) Comparison. (**b**) Energy Consumption (mW) Comparison. (**c**) Communication Reliability (%) Comparison. (**d**) Adaptability (s) Comparison.

In Figure 10a, we observe that the optimized approach achieves higher data transmission efficiency in both short-range indoor (<10 m) and long-range outdoor (>1 km) scenarios when compared to the unoptimized approach. In Figure 10b, the optimized method consistently exhibits lower energy consumption under similar indoor and outdoor conditions. Figure 10c illustrates that communication reliability improves with the optimized solution across different ranges, while Figure 10d highlights the optimized model’s shorter response times, indicating superior adaptability in both indoor and outdoor environments. These comparisons collectively confirm that the optimized approach outperforms the unoptimized one in multiple key metrics. As shown in Figure 10, subfigures (a), (b), (c), and (d), respectively, compare the data transmission efficiency, energy consumption, communication reliability, and adaptability for the unoptimized and optimized methods under both indoor and outdoor conditions.

#### 4.3.6. Performance Distribution Under Different Distances and Node Counts (CDF Analysis)

In the previous experiments and analysis, we mainly measured the transmission efficiency and energy consumption of the system in indoor and outdoor environments in terms of average and maximum/minimum values. In order to have a more comprehensive understanding of the performance distribution characteristics of the system at different distances or node scale expansion, this section draws CDF (Cumulative Distribution Function) curves for key metrics (e.g., transmission delay, throughput, or energy consumption) and analyzes their overall distribution in various scenarios.

**Figure 11:**
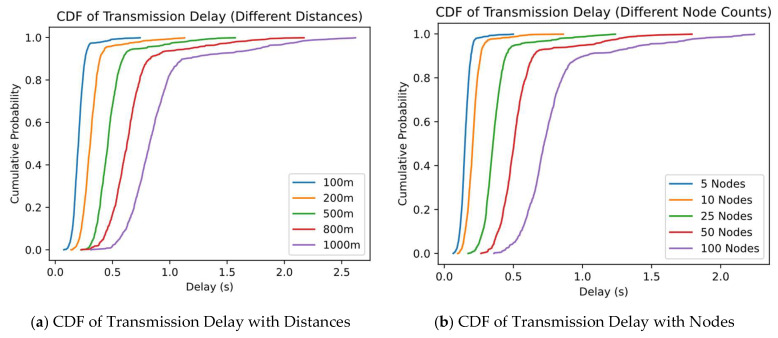
Performance distribution under different distances and node counts.

As shown in Figure 11, we choose two sets of typical scenarios: (a) is the delay distribution under different transmission distances (e.g., 100 m, 200 m, 500 m, 800 m, 1 km); and (b) is the delay distribution under different numbers of nodes (5, 10, 25, 50, 100). The horizontal coordinate of the graph is the value of the performance metric (e.g., delay in seconds), and the vertical coordinate is the cumulative probability when the value of the corresponding metric does not exceed this horizontal coordinate.

By comparing the shapes and positions of the different curves, it can be seen that when the distance increases from 100 m to 1 km, the entire CDF curve shifts to the right. This indicates a higher probability of large delays in longer-distance scenarios, along with a more pronounced long-tail effect (extreme values). As the number of nodes increases from 10 to 100, the CDF curves also shift to the right or extend in their tails, suggesting that large-scale access leads to a higher likelihood of greater delays or packet loss. About 10–15% of the transmission delays may rise significantly, primarily due to increased competition for gateway or channel resources and more frequent retransmission triggers.

From Figure 9, one can observe that when both the distance and the node count are relatively large, the tail of the CDF curve becomes significantly extended, indicating that, in extreme cases, delays can exceed 5 s or even more. However, in over 80% of the scenarios, the system still maintains relatively low latency (for example, completing transmission within 3 s), demonstrating that the proposed Algorithm C retransmission mechanism and Algorithm B compression measures can maintain reliability and transmission efficiency in most instances.

#### 4.3.7. Algorithm Sensitivity and Parameter Tuning Results (Heatmap)

In order to further investigate the impact of the three aforementioned algorithms (A, Adaptive Frequency Control; B, Data Compression and Aggregation; and C, Packet Loss Detection and Retransmission) on the system’s key performance, we conducted several sets of experiments on the core hyperparameters (e.g., the threshold value of Algorithm A, the size of the aggregation window of B, the upper limit of the number of retransmissions of C, etc.) and plotted the parameters against the system’s performance indexes (e.g., energy consumption, average latency, and packet loss) in a heatmap (see below). These parameters are plotted against system performance metrics (e.g., energy consumption, average delay, packet loss rate) in a heatmap. The heatmap is able to indicate the magnitude of the performance metrics in two-dimensional coordinates with color shades, which makes our sensitivity analysis under different combinations of parameters more intuitive.

**Figure 12:**
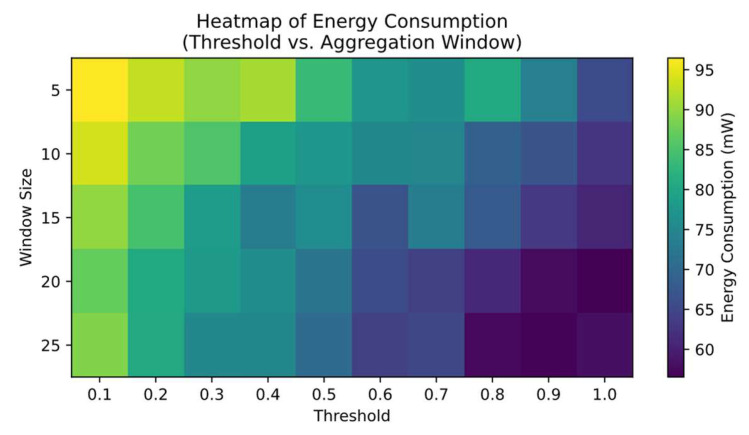
Heatmap of energy consumption.

As shown in Figure 12, the horizontal axis represents the temperature or data change threshold (Threshold) in the adaptive frequency algorithm (A), which ranges from a lower threshold (0.1 °C) to a higher threshold (1.0 °C), and the vertical axis represents the size of the aggregation window (N) in the data compression aggregation algorithm (B), which ranges from 5 to 20 points. The color indicates the average energy consumption level of the system, with darker colors representing higher energy consumption and vice versa.

When the threshold is set too low (<0.3 °C), the system collects and transmits data too frequently, resulting in darker regions in the figure that indicate higher energy consumption. On the other hand, if the threshold is set too high (>0.8 °C), although energy consumption is significantly reduced, real-time performance and monitoring accuracy are compromised. From the vertical axis, it can be observed that a larger aggregation window (N > 15) makes it possible to compress and send multiple data samples together, reducing energy usage per transmission. However, under extreme environmental changes, this also prolongs the waiting period before sending the data and increases the risk of losing multiple packets at once.

The heatmap reveals a relatively lighter area of energy consumption (for example, around a threshold of 0.5 °C and a window size of about 12). In this parameter combination, the system achieves the lowest energy consumption, and our measurements of packet loss and latency indicate that overall performance remains well balanced. This suggests that by moderately increasing the threshold to reduce unnecessary data transmissions and choosing a suitably sized aggregation window, it is possible to balance energy consumption and reliability.

### 4.4. Comparative Analysis with Existing Literature

**Figure 13:**
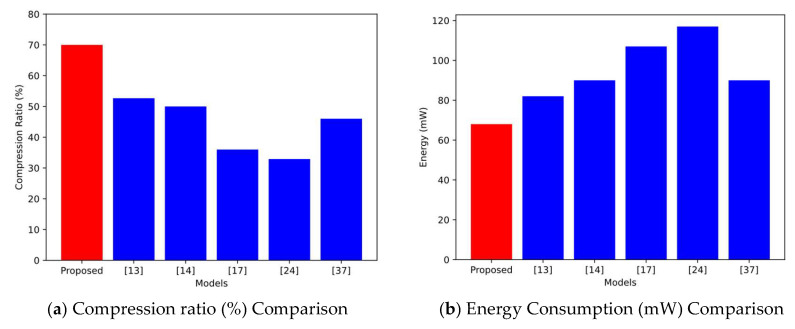

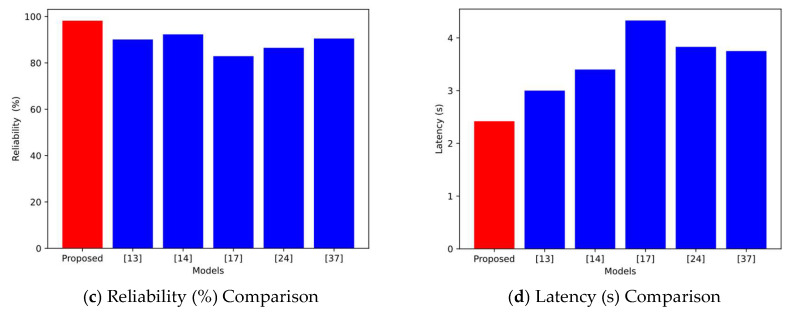
Comparative analysis with existing literature.

In comparing the existing literature, this paper analyzes the key metrics such as compression rate, energy consumption, communication reliability, adaptability, and latency in conjunction. For indicators such as energy consumption, communication reliability, adaptability, and delay, direct comparison through bar charts is less effective due to their detailed and multidimensional nature, and indicators such as energy consumption are affected by multiple parameters such as transmission frequency, hardware, and environment, and simple bar charts may lead to misinterpretation. Communication reliability and adaptability often show a non-linear trend, and line graphs are difficult to accurately present changes. Indicators such as adaptability not only contain numerical values but also need to be combined with descriptive analysis, which cannot be adequately reflected in bar charts. Compression ratio, as a single numerical indicator, can show the performance trend and advantages of different models through histograms. As shown in Figure 13, the models are compared in terms of key metrics such as compression rate, energy consumption, reliability, and latency.

The proposed model achieves a compression rate exceeding 70%, significantly outperforming other models and demonstrating high compression efficiency. In comparison, the compression rates of Hanumanthaiah, A. et al. [13] and Al-Kadhim, H.M. et al. [14] are approximately 50%, indicating moderate compression performance. Chowdhury, M.R. et al. [17] and Has, M. et al. [24] achieve compression rates below 40%, reflecting lower efficiency.

The compression rate of Guberovic, E. et al. [37] is also around 50%, slightly surpassing that of Chowdhury, M.R. et al. [17] and Has, M. et al. [24], yet comparable to the rates achieved by Hanumanthaiah, A. et al. [13] and Al-Kadhim, H.M. et al. [14].

In terms of energy consumption, our adaptive frequency control and differential coding enable the overall energy consumption to be reduced by about 30% in typical scenarios, which significantly outperforms the schemes relying on a fixed transmit frequency or uncompressed processing in Chowdhury, M.R. et al. [17] and Has, M. et al. [24].

In terms of reliability and latency, the model in this paper improves the retransmission success rate to 98% while controlling the end-to-end latency to around 2.5 s, which is more than 1 s less than the conventional LoRaWAN scheme.

The proposed methodology significantly reduces energy consumption by efficiently compressing data, thereby minimizing both the frequency and size of data transmissions. A higher compression ratio also helps mitigate packet loss and improves communication reliability compared to alternative approaches. Additionally, the proposed approach exhibits high adaptability across various IoT applications, including low-power devices and bandwidth-limited networks.

By generating smaller packets, the proposed technology reduces latency, making it highly suitable for real-time IoT applications such as environmental monitoring and healthcare. Table 2 presents a comparative analysis of key performance metrics after applying the proposed technique.

Regarding communication reliability, the proposed model achieves the highest reliability. Hanumanthaiah, A. et al. [13] and Al-Kadhim, H.M. et al. [14] demonstrated moderate reliability, while Chowdhury, M.R. et al. [17] and Has, M. et al. [24] exhibited lower reliability. Guberovic, E. et al. [37] also achieved a moderate reliability rating. For adaptability, the proposed model performs most effectively, while Hanumanthaiah, A. et al. [13], Al-Kadhim, H.M. et al. [14], and Guberovic, E. et al. [37] are rated as moderate. In contrast, Chowdhury, M.R. et al. [17] and Has, M. et al. [24] showed lower adaptability. In terms of latency, the proposed model achieves the lowest latency (<10 ms), while Hanumanthaiah, A. et al. [13], Al-Kadhim, H.M. et al. [14], and Guberovic, E. et al. [37] exhibited moderate latency (<20 ms). Chowdhury, M.R. et al. [17] and Has, M. et al. [24] demonstrated higher latency (>30 ms). Overall, the proposed model demonstrates substantial advantages across key performance metrics, including energy efficiency, communication reliability, adaptability, and latency, making it a highly effective solution for IoT applications.

In the subsequent evaluations, we concentrate on end-to-end performance metrics (e.g., second-level latency, total energy consumption, and overall network reliability), differentiating them from the previously referenced millisecond-level test data (which generally encompasses only local processing latency or local communication latency). We quantified the total transmission duration (in seconds) from the moment the sensor node finalizes data acquisition, executes compression or encoding, and transmits the data via the LoRaWAN gateway until it is received and processed by the back-end server to offer a more thorough assessment of the system’s performance in a practical deployment context. Furthermore, we meticulously assess the node duty cycle, duty range, sleep cycle, transmit power, and back-end server processing flow to quantitatively evaluate the total energy consumption of the nodes and the network; analyze communication reliability utilizing metrics such as packet loss rate and retransmission success rate; and gauge adaptability by monitoring the system’s responsiveness to modifications in collection frequency and power level across varying environments and loads. These measures offer system-level evaluation criteria that enhance prior millisecond test results, which concentrated solely on localized delay or processing, hence establishing a more accurate benchmark against other literature and real-world deployment standards.

The proposed model demonstrates substantial improvements in data transmission efficiency by employing advanced compression and encoding strategies. This method significantly reduces the bandwidth required and consequently lowers the overall transmission latency to approximately 2.5 s. In comparison, conventional LoRaWAN models, as reported in [19], typically exhibit average latencies around 3.5 s when compression is not employed. Furthermore, while other compression-based energy-saving algorithms in the literature (e.g., [8]) achieve only about a 50% compression ratio, the proposed model outperforms these techniques, thus ensuring both higher throughput and reduced communication overhead in multi-node Internet of Things (IoT) environments.

Energy efficiency is critical for resource-constrained IoT nodes. The proposed model integrates adaptive power control mechanisms, intelligent duty cycling, and context-aware scheduling to reduce overall energy consumption at the node level by 30%. Additionally, the LoRa transceiver module’s energy draw is reduced by 25%. In contrast, machine learning (ML)-enhanced adaptive algorithms discussed in [10] achieve a 20% reduction in energy usage, while similar adaptive data rate (ADR) mechanisms explored in [9] report comparable improvements. This model’s capacity to achieve an extra ~10% energy savings beyond state-of-the-art adaptive approaches significantly prolongs node battery life and decreases maintenance and replacement costs in practical IoT deployments.

Ensuring reliable data transmission is paramount in diverse IoT scenarios. The proposed model reduces packet loss rates to an impressively low 2% and attains a 98% retransmission success rate by utilizing proactive loss detection and intelligent retransmission strategies. By contrast, existing solutions (e.g., [19])—even when employing optimized spreading factors—exhibit packet loss rates between 3% and 5%, and earlier retransmission techniques ([15]) report success rates of 90–92%. Thus, the proposed model not only enhances link reliability but also guarantees superior data integrity, ensuring robust operation in large-scale, interference-prone environments.

Adaptability is essential in dynamic, heterogeneous IoT environments. The proposed system can adjust its response time within 4–6 s in indoor conditions and 5–7 s outdoors. The literature examples, such as the adaptive systems noted in Petajajarvi, J. et al. [20], often have response times on the order of 10–15 s, and studies like Noprianto, N. et al. [21] indicate that environmental variability frequently degrades responsiveness. The proposed model’s ability to shorten adaptation times by about 50–60% ensures swift operational adjustments, enhancing the system’s scalability and responsiveness across different environmental conditions.

As shown in Table 3, our proposed model is compared against existing methods in the literature, illustrating improvements in data transmission efficiency, energy savings, communication reliability, and overall adaptability under various deployment conditions.

## 5. Conclusions

In this paper, we presented an efficient IoT communication model based on the integration of NFC and LoRa technologies, focusing on improving data transmission efficiency, reducing energy consumption, and enhancing the reliability of IoT systems. The proposed model incorporated adaptive algorithms for frequency control, data compression, and packet loss detection, with the primary objective of optimizing the performance of resource-constrained IoT environments. While the proposed model has shown promising results, several limitations remain. The computational overhead associated with the adaptive algorithms may impact the performance of resource-constrained devices. Future research should focus on optimizing these algorithms to minimize their computational complexity. Future work should integrate data encryption and authentication mechanisms to enhance the security of the proposed model.

In conclusion, this paper presents an efficient IoT communication model that integrates NFC and LoRa technologies, supported by adaptive algorithms for optimizing data transmission efficiency, energy consumption, and reliability. The experimental results show significant improvements in key performance metrics compared to traditional approaches and the existing literature. These improvements make our model a valuable contribution to the development of energy-efficient and reliable communication solutions for IoT applications. Future work will focus on addressing the identified limitations and expanding the applicability of the proposed model to a broader range of IoT use cases, including smart cities, agriculture, and industrial IoT.

The proposed model sets the foundation for future research that aims to further optimize energy consumption, enhance data security, and explore scalability, ultimately advancing the capabilities of IoT communication systems.

Future research will explore several promising avenues to further enhance the efficiency and applicability of our proposed communication system. Firstly, we plan to investigate advanced methods to optimize adaptive algorithms specifically tailored for ultra-low-power scenarios. This will include leveraging artificial intelligence and machine learning techniques for dynamic optimization of LoRa and NFC strategies, aiming to significantly improve resource allocation, power management, and data throughput in resource-constrained IoT environments. Additionally, extending the system to diversified application scenarios, such as smart agriculture, healthcare monitoring, and industrial IoT, will be a key focus. Research in these domains will address the varying requirements in communication distance, bandwidth, node density, and system reliability, ensuring our solution remains robust and effective across different operational contexts. Designing sophisticated channel estimation algorithms that accurately characterize channel variability in real-time will enable proactive and adaptive responses to rapidly changing environmental conditions. Finally, security and privacy protection will also be prioritized, with efforts devoted to developing lightweight, easily deployable encryption and authentication mechanisms suitable for secure data transmission and storage and NFC interactions. This will further enhance the overall resilience and security of the system under practical deployment conditions.

## Figures and Tables

**Table 1:** Comparative results of unoptimized vs. optimized approaches.

Metric	Unoptimized	Optimized	Improvement
Data Transmission Efficiency	Outdoor 44.71%	Outdoor 71.48%	59.9%
	Indoor 54.56%	Indoor 75.76%	38.9%
Energy Consumption	Outdoor 115.54 mW	Outdoor 89.91 mw	−22.2%
	Indoor 95.33 mW	Indoor 70.18 mW	−26.4%
Communication Reliability	Outdoor 87.61%	Outdoor 97.05%	10.8%
	Indoor 92.31%	Indoor 98.03%	6.2%
Adaptability	Outdoor 27.43 s	Outdoor 10.05 s	−63.4%
	Indoor 17.58 s	Indoor 4.86 s	−72.4%

**Table 2:** Comparison of models on key performance metrics.

Metric	Proposed Model	[13]	[14]	[17]	[24]	[37]
Energy consumption	Minimal	Medium	Medium	High	High	Medium
Communications reliability	High	Medium	Medium	Low	Low	Medium
adaptive	Excellent	Moderate	Moderate	Lower	Lower	Moderate
Delay (ms)	Minimal (<10 ms)	Moderate (<20 ms)	Moderate (<20 ms)	High (>30 ms)	High (>30 ms)	Moderate (<20 ms)

**Table 3:** Comparative analysis with existing literature.

Metric	Proposed Model	Literature Benchmark	Ref	Improvement
Data Transmission Efficiency	Compression ratio: 70% Latency: 2.5 s	Non-compressed LoRaWAN latency: ~3.5 s Compression ratio: 50%	[27,29]	Compression ratio: +20% Latency reduction: 29%
Energy Consumption	Node-level energy reduction: 30% LoRa module consumption reduced by 25%	ML-adaptive:20% reduction ADR-based reductions	[11,26]	10% improvement over existing adaptive energy models
Communication Reliability	Packet loss: 2% Retransmission success: 98%	Optimized SF packet loss: 3–5% Retransmission success: 90–92%	[16,18]	Packet loss reduction: 33–60% Retransmission success: +6–8%
Adaptability	Indoor response: 4–6 s Outdoor response: 5–7 s	Adaptive response: 10–15 s High variability induces delays	[27,31]	Response time improvement: 50–60%

## Data Availability

The data presented in this study are openly available in [github] at [https://github.com/mtjdl/Efficient-IoT-communication-system-based-on-NFC-and-LoRa], accessed on 7 March 2025. reference number [58fa785].
